# Early mechanical ventilation in patients with Guillain-Barré syndrome at high risk of respiratory failure: a randomized trial

**DOI:** 10.1186/s13613-020-00742-z

**Published:** 2020-09-30

**Authors:** Marie-Anne Melone, Nicholas Heming, Paris Meng, Dominique Mompoint, Jerôme Aboab, Bernard Clair, Jerôme Salomon, Tarek Sharshar, David Orlikowski, Sylvie Chevret, Djillali Annane

**Affiliations:** 1 Respiratory, Thoracic Oncology and Respiratory Intensive Care Department, Rouen University Hospital, Rouen, France; 2 Medical Intensive Care Unit, Raymond Poincaré Teaching Hospital, 104 boulevard Raymond Poincaré, 92380 Garches, France; 3 Radiology Department, Raymond Poincaré Teaching Hospital, Garches, France; 4 Biostatistic Team, Saint Louis Hospital, Paris, France; 5 Infectious Diseases Department, Raymond Poincaré Teaching Hospital, Garches, France

**Keywords:** Guillain-Barré syndrome, Acute respiratory failure, Intubation, Mechanical ventilation, Aspiration

## Abstract

**Introduction:**

About 30% of patients with Guillain-Barré syndrome become ventilator dependent, of whom roughly 75% develop pneumonia. This trial aimed at assessing the impact of early mechanical ventilation (EMV) on pneumonia occurrence in GBS patients. We hypothesize that EMV will reduce the incidence of pneumonia.

**Methods:**

This was a single centre, open-label, randomized controlled trial performed on two parallel groups. 50 intensive care unit adults admitted for Guillain-Barré syndrome and at risk for acute respiratory failure. Patients were randomized to early mechanical ventilation via face-mask or endotracheal intubation owing to the presence or absence of impaired swallowing (experimental arm), or to conventional care (control arm). The primary outcome was the incidence of pneumonia up to intensive care unit discharge (or 90 days, pending of which occurred first).

**Findings:**

Twenty-five patients were randomized in each group. There was no significant difference between groups for the incidence of pneumonia (10/25 (40%) vs 9/25 (36%), *P* = 1). There was no significant difference between groups for the time to onset of pneumonia (*P* = 0.50, Gray test). During follow-up, there were 16/25 (64%) mechanically ventilated patients in the control group, and 25/25 (100%) in the experimental arm (*P* < 000·1). The time on ventilator was non-significantly shorter in the experimental arm (14 [7; 29] versus 21.5 [17.3; 35.5], *P* = 0.10). There were no significant differences between groups for length of hospital stay, neurological scores, the proportion of patients who needed tracheostomy, in-hospital death, or any serious adverse events.

**Conclusions:**

In the present study including adults with Guillain-Barré syndrome at high risk of respiratory failure, we did not observe a prevention of pneumonia with early mechanical ventilation. Trial registration: ClinicalTrials.gov under the number NCT00167622. Registered 9 September 2005, https://clinicaltrials.gov/ct2/show/NCT00167622?cond=Guillain-Barre+Syndrome&cntry=FR&draw=2&rank=1

## Background

Guillain-Barré syndrome (GBS) is a common cause of non-traumatic acute paralysis with an incidence of about 0·62–2·66 per 100 000 person-years in Europe and North America [1, 2]. Patients may develop acute respiratory failure as a result of progressive weakness of inspiratory and expiratory muscles, and of bulbar dysfunction [3, 4]. In practice, about 30% of GBS patients become ventilator dependent, with subsequent increase risk of death [5–9]. The optimal timing for mechanical ventilation in patients with GBS remains controversial [10–12]. On one hand, emergency intubation may trigger cardiovascular dysautonomia which may precipitate death [13]. On the other hand, several factors may identify a group of patients at high risk of respiratory failure. First, more than 80% of GBS patients admitted to the ICU within 7 days of motor-deficit onset, being unable to lift the head, and whom forced vital capacity was of less than 60% of predicted value, eventually presented with acute respiratory failure requiring mechanical ventilation [14]. Second, early swallowing impairment is present in approximately 80% of GBS patients admitted to the ICU, and is associated with substantial increase in the risk of acute respiratory failure, endotracheal intubation and mechanical ventilation [15]. The incidence of ventilator-associated pneumonia in GBS patients is about 75% and is more likely to occur with delayed mechanical ventilation [16]. This trial aimed at assessing the impact of early mechanical ventilation (EMV) on pneumonia occurrence in GBS patients. We hypothesize that EMV will reduce the incidence of pneumonia.

## Materials and methods

### Study design

The Ethics Committee (Comité de Protection des Personnes) of Saint-Germain-en-Laye, France, approved the trial protocol. Participants or their legally authorized next of kin provided written informed consent before inclusion whenever possible. Otherwise, deferred written informed consent was obtained from patients. This investigator-led trial was publicly funded. This single-center, controlled trial, conducted with two parallel groups aimed to evaluate the impact of early initiation of mechanical ventilation on pneumonia occurrence in GBS patients. All authors had full and independent access to all data, and vouch for the integrity, accuracy, and completeness of the data and analysis, and for the adherence to study protocol.

The trial was registered at ClinicalTrials.gov under the number NCT00167622, before inclusion of the first patient.

### Participants

Adults patients admitted to the intensive care unit with Guillain-Barré syndrome were enrolled in the study if they met all the following risk factors for endotracheal intubation, (1) time from onset to admission of less than 7 days, (2) inability to lift the head, and (3) forced vital capacity of < 60% of predicted. Patients were excluded when they had either criterion of the presence of respiratory distress signs, PaC02 greater than 6.4 kPa, PaO_2_ of less than 7.5 kPa, and forced vital capacity of less than 20% of predicted value or less than 15 mL/kg of body weight. Others exclusion criteria were an age below 18 years old, altered consciousness (Glasgow coma score < 8), pregnancy, hemodynamic instability, pre-existing pneumonia.

### Randomization and masking

Patients were randomized 1:1 to early mechanical ventilation or to physiotherapy and oxygen whenever needed (control group). Randomization was based on computer-generated variable block sizes stratified according to presence or not of swallowing difficulties. The randomization sequence was prepared by the study statistician who did not take part in randomization. Allocation was concealed by means of opaque sealed envelopes until qualifying patients were consented and ready for mechanical ventilation. Health care professionals taking part in the intervention were aware of the treatment assignment, because they were responsible for implementing the designated respiratory management. However, assessors of the primary endpoint were fully blinded to treatment. Investigators were unaware of all data until the trial concluded.

### Interventions

In all aspects of treatment, except regarding respiratory management, all patients in both groups of the trial, were treated according to the most recent guidelines for the management of Guillain-Barré syndrome, including immunotherapy [17]. Intravenous immunoglobulins were chosen in case of suspected infection, they were perfused at 0.5 g/kg daily for 5 days. Plasma exchange were realized at number of 4 amounting to 200 to 250 mL/kg with albumin 20% or crystalloids or colloids solution. All patients with suspected hospital acquired pneumonia were treated following identification of pathogens. In patients with sepsis, empiric antibiotherapy was initiated including one of piperacillin–tazobactam, cefepime, or imipenem, meropenem, owing to the intensive care unit bacterial ecology at the time the trial was conducted.

In the control arm, patients benefited from physiotherapy, including active clearance of bronchial secretion and oxygen via a face mask whenever needed. In this group of patients, mechanical ventilation with endotracheal intubation was not permitted unless acute respiratory failure occurred defined by the presence of respiratory distress signs, PaC02 greater than 6.4 kPa, arterial oxygen tension of less than 7.5 kPa, and forced vital capacity of less than 20% of predicted value or less than 15 mL/kg of body weight.

In the experimental arm, mechanical ventilation was initiated immediately after randomization either via a full-face mask or endotracheal intubation owing to the presence or absence of impaired swallowing (early mechanical ventilation—EMV). If patients underwent acute respiratory failure or new swallowing impairment, they received endotracheal intubation. They benefited daily from physiotherapy including active clearance of bronchial secretion with the same ex-insufflation maneuver as the control arm.

### Investigated parameters

At baseline, we systematically recorded age, gender, Knaus disability scale [18], SAPSII [19], MRC sumscore [20], the presence of facial palsy or of swallowing impairment, spirometric data (forced vital capacity (mL and percent of predicted value), inspiratory pressure and expiratory pressure (cm of water) and arterial blood-gas.

We collected daily over the first week and weekly up to day 60 post-randomization, vital signs, core temperature (°C), biological data (liver enzymes, white blood cells count, PaO_2_/FiO_2_ ratio), bacteriological data (bloodstream culture and culture of sampling from any suspected site of infection) and immunotherapy (plasma exchange or intravenous immunoglobulin). We evaluated daily swallowing impairment by clinical deglutition test including the Volume-Viscosity Swallow Test.

### Outcomes

Primary outcome was the incidence of first episode ventilator-acquired pneumonia up to ICU discharge (or 90 days, pending which occurred first).

Secondary outcomes included time to onset of pneumonia, time to and on mechanical ventilation, length of hospital stay, requirement for tracheostomy, mortality and any serious adverse events (septic shock, hepatic failure, renal failure, intravascular coagulopathy, acute respiratory distress syndrome).

### Definitions

The definition for ventilator-acquired pneumonia was adapted from ATS/IDSA criteria [21] and required the new onset, i.e. > 48 h following randomization, of lung infiltrate or progressive radiographic infiltrate, a temperature higher than 38.3 °C, purulent secretions, leukocytosis or leukopenia (white blood cell count higher than 12,000/mm^3^ or lower than 4000/mm^3^), and altered oxygenation.

If pneumonia was suspected, broncho-alveolar lavage or protected brush bronchial samples were performed. Microbiological studies of the respiratory samples were considered positive if the number of colony-forming units (CFUs) for any isolated pathogen was greater than 10^4^/ml.

The evaluation of the presence or absence of ventilator-acquired pneumonia was made by three independent adjudicators (an infectious disease physician, a radiologist and a pneumologist) who were blinded to study treatments.

### Statistical analysis

The sample size was based on of the estimation that ventilator-acquired pneumonia will occur in 75% of patients with Guillain-Barré syndrome and dependent on mechanical ventilation [16]. Then, to detect a 50% absolute reduction in the incidence of pneumonias with a 2-sided significance level of 0·05 and power of 80%, we calculated that 25 subjects should be included per study arms, for a total of 50 participants.

The statistical analysis was performed according to the intent-to-treat principle, and once after all included patients reached the last time point of follow-up. Statistical data are reported as median [interquartile] for continuous variables and number (percentage) for qualitative variables.

The time to onset of pneumonia up to ICU discharge (or 90 days, pending which occurred first) was compared using the Gray’s test. Others outcomes were compared using the Wilcoxon signed-rank test or Fisher exact test as appropriate.

The open-source R software version 2.15.2 (2012-10-26), and LATEX, on a platform i386−w64−mingw32 was used for all statistical tests. Figures and tables were automatically created with *Sweave*.

### Role of funding source

The trial was funded by Assistance Publique Hôpitaux de Paris. The funder had no role in designing the trial, collecting or analyzing the data, data interpretation, writing the manuscript, or decisions related to submission for publication.

## Results

### Study population

**Fig. 1:**
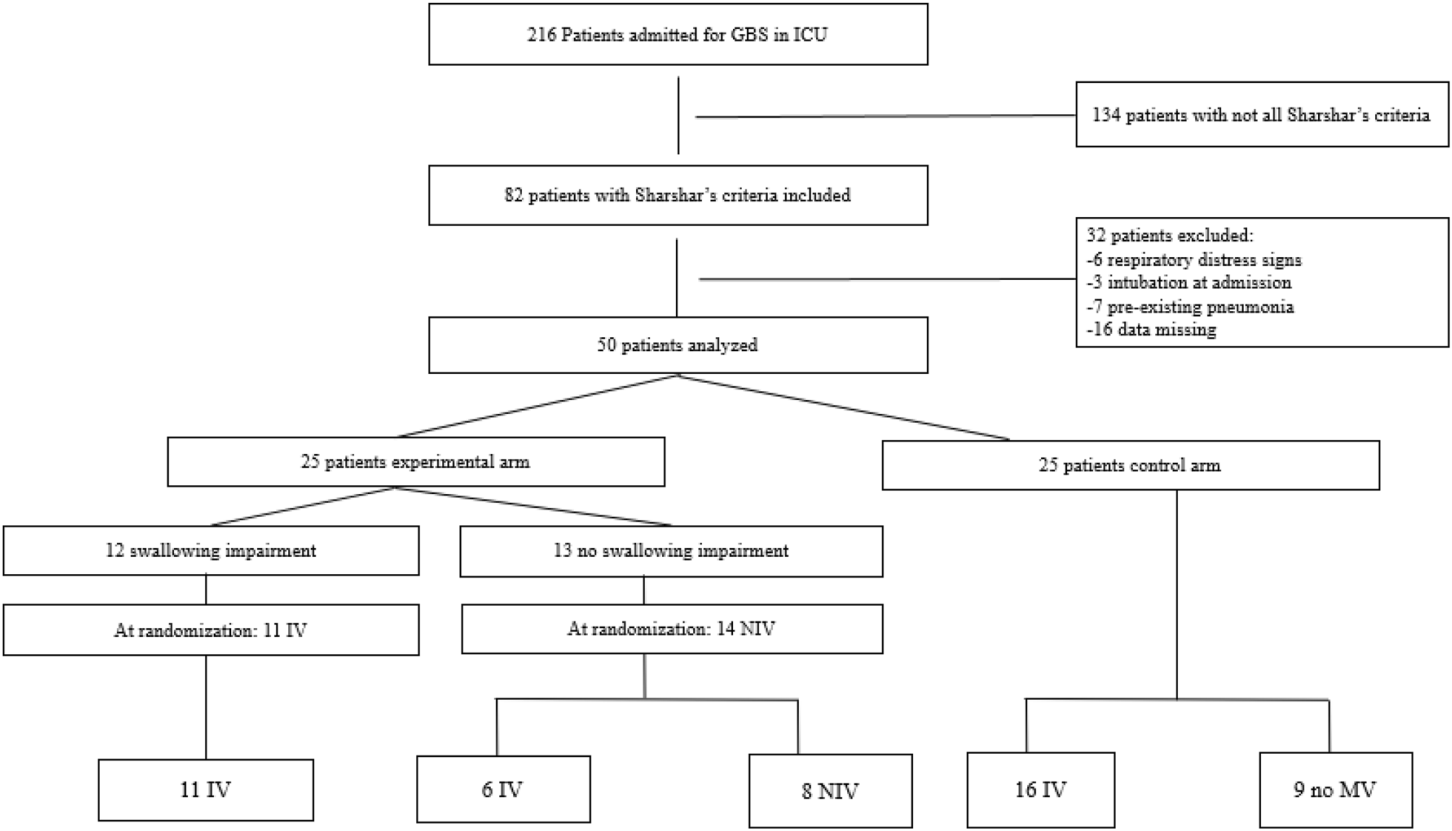
Flowchart. IV: invasive ventilation; NIV: non-invasive ventilation; no MV: no mechanical ventilation

A total of 50 patients (25 in each treatment arm) were recruited (Fig. 1) from December 2004 to November 2008. Patients’ median age was 57 [44–69] years, and 56% were men (Table 1). Swallowing impairment was present in 12/25 (48%) patients in the EMV group and in 13/25 (52%) patients in the control group. In the EMV group, all patients were mechanically ventilated including 11/25 (44%) requiring invasive mechanical ventilation. In experimental arm, one patient with swallowing impairment was treated by non-invasive ventilation. There was no evidence for clinically relevant differences in baseline characteristics between groups (Table 1). Seven patients did not receive any immunotherapy, 28 were treated with intravenous immunoglobulin (0.5 g per kilogram of body weight daily for 5 days), 10 with plasma exchange (1 session every over day for a total of 4) and 5 patients received both treatments (Table 1).

**Table 1:** Baseline characteristics

	Control group *N* = 25	Experimental group *N* = 25	All patients *N* = 50	
Age (years)	57 [46–70]	56 [39–67]	57 [44–69]	0.47
Male	13 (52)	15 (60)	28 (56)	0.58
Knaus score				0.21
A	20 (80)	16 (64)	36 (72)	
B	4 (16)	7 (28)	11 (22)	
C	1 (4)	1 (4)	2 (4)	
D	0 (0)	1 (4)	1 (2)	
SAPSII	21 [17–33]	30 [17–34]	26 [17–34]	0.58
MRC sumscore (/50)	38 [30–42.75]	28 [22.5–37]	33 [25–40]	0.043
Facial palsy	5 (22)	9 (39)	14 (30)	0.21
Swallowing impairment	13 (52)	12 (48)	25 (50)	1.00
FVC (ml)	1460 [1105–2335]	1726 [1206–1888]	1600 [1184–2075]	0.84
FVC (%pred)	51 [41.25–58]	47 [40–54]	49 [40–56]	0.22
MIP (cmH20)	30 [20–43]	45 [26–50]	40 [21–50]	0.21
MEP (cmH20)	32.5 [28–43·25]	50 [35–57.5]	40 [30–52.5]	0.12
FiO_2_ (%)	21 [21–60]	30 [21–40]	25·5 [21–43]	0.61
PaCO_2_ (kPa)	4.98 [4.53–5.52]	5.31 [4.56–5.6]	5.06 [4.54–5.6]	0.66
PaO_2_ (kPa)	10.95 [9.43–12.45]	11.8 [9.63–12.3]	11.55 [9.50–12.35]	0.54
SatO_2_ (%)	96.6 [95.0–98.6]	97 [93.3–98.5]	97 [94.6–98.2]	0.84
pH	7.43 [7.39–7.46]	7.43 [7.4–7.45]	7.43 [7.4–7.46]	0.74
HCO_3_-(mmol/L)	25·1 [22.92–26.92]	25 [23.35–27.4]	25.1 [23–27]	0.75
No immunotherapy	5 (20)	2 (8)	7 (14)	0.38
IvIg	11 (44)	17 (68)	28 (56)	
Plasma exchange	6 (24)	4 (16)	10 (20)	
IvIg and plasma exchange	3 (12)	2 (8)	5 (10)	

Data are expressed as median and interquartile range for continuous variables, and as number and percentage for categorical variables

*SAPSII* simplified acute physiology score II, *MRC sumscore* Medical Research Council sumscore, *FVC* forced vital capacity, *MIP* maximal inspiratory pressure, *MEP* maximal expiratory pressure, *PaCO*_*2*_ arterial pressure of carbon dioxide, *PaO*_*2*_ arterial pressure of oxygen, *SatO*_*2*_ transcutaneous saturation of oxygen, *IvIg* Intravenous Immunoglobulin

### Primary outcome

**Fig. 2:**
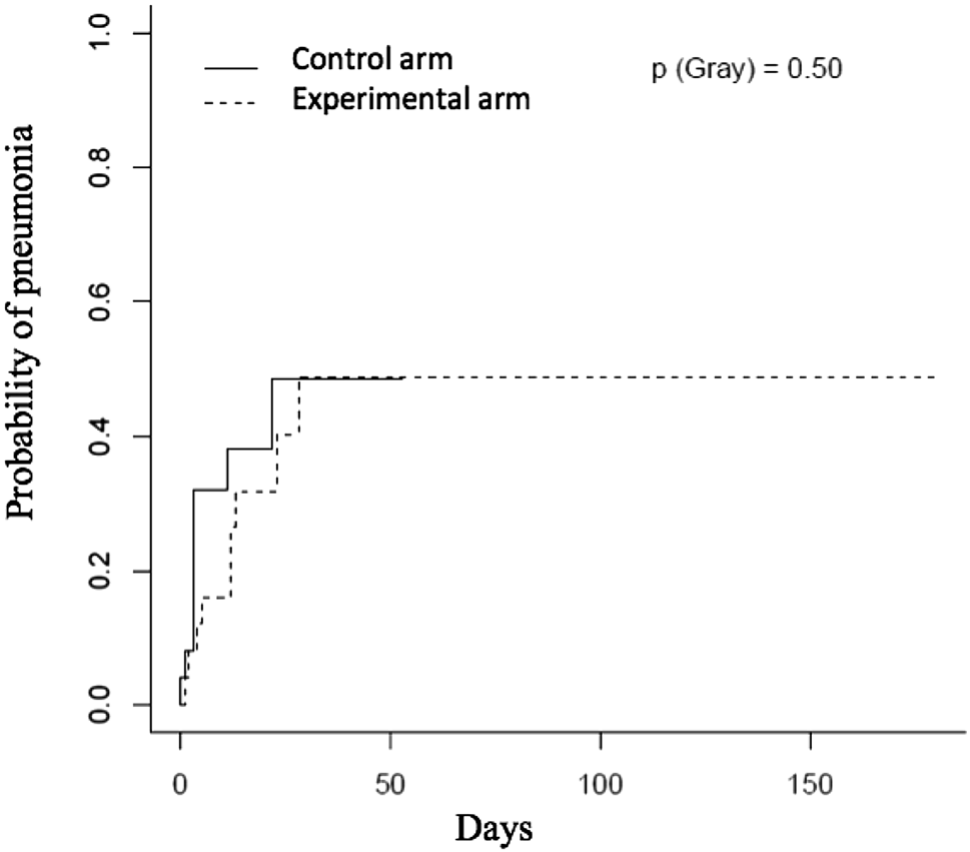
Time to the first episode of hospital acquired pneumonia

A total of 19/50 (38%) patients developed at least one episode of ventilator-acquired pneumonia, 9/25 (36%) in the EMV group and 10/25 (40%) in the control group (*P* = 1.00). Twenty-nine episodes of VAP occurred in 19/50 patients (Table 2). Only, 1/8 (12.5%) developed hospital-acquired pneumonia in NIV group. At 28 days, the probability of onset of pneumonia was 49·6% [30.9–68.4]. There was no significant difference between groups regarding the time to the first episode of ventilator-acquired pneumonia (*P* = 0.50) (Fig. 2). Microbial data are provided in Additional file 1: Table S1.

**Table 2:** Primary outcome—distribution of ventilator-acquired pneumonia across randomization groups

Episodes of ventilator-acquired pneumonia	All patients *N* = 50	Experimental group *N* = 25	Control group *N* = 25	*P *value
At least 1 episode	19 (38)	9 (36)	10 (40)	1.00
1 episode	10 (20)	6 (24)	4 (16)	
2 episodes	8 (16)	2 (8)	6 (24)	
3 episodes	1 (2)	1 (4)	0 (0)	

Data are expressed as number (percentage)

### Secondary outcomes

**Fig. 3:**
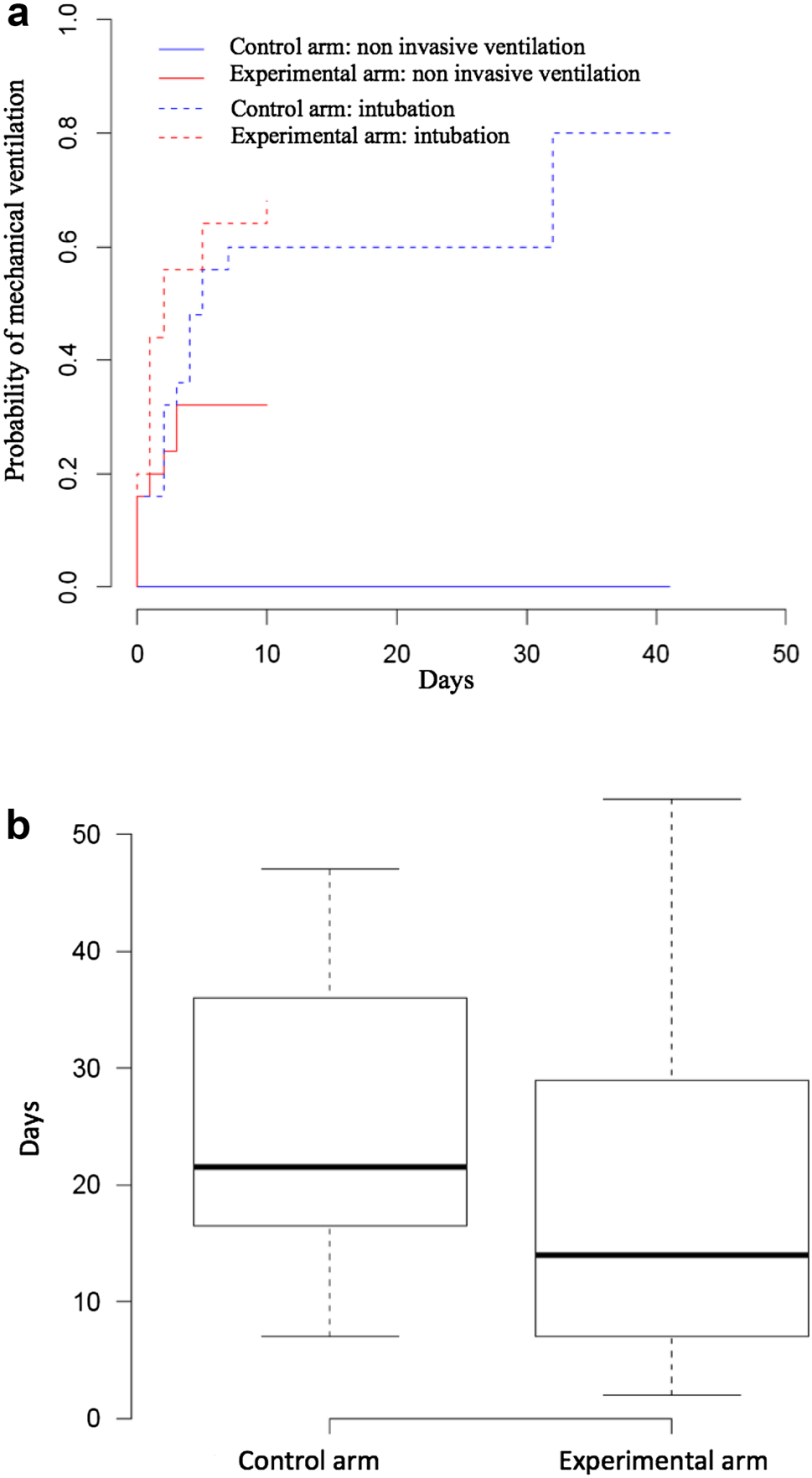
Cumulative incidence and time on mechanical ventilation. **a** Cumulative incidence of mechanical ventilation. **b** Time on mechanical ventilation

There were 16/25 (64%) patients who needed mechanical ventilation (in all cases invasive mechanical ventilation) and 25/25 (100%) patients (8 non-invasively and 17 were intubated) in the experimental arm (*P* < 000.1) (Table 3). In the EMV group, of the 17 intubated patients, 6 were initially on non-invasive ventilation. The median time to initiation of mechanical ventilation was 1 [0–2] days in the experimental arm, and 5 [2–9] days in the control group (Fig. 3a). The time on mechanical ventilation was 14 [7–29] days in the experimental arm and 21.5 [17.3–35.5] days in the control arm (*P* = 0.10) (Fig. 3b). Overall, 10/50 (20%) patients required a tracheostomy; 4/25 (16%) patients in the experimental arm and 6/25 (24%) in the control arm (*P* = 0.55). In the experimental arm, the median time to tracheostomy was 37 [32.5–44] days in the experimental arm and 36 [34–38] days in the control arm (see Additional file 1: Figure S1).

**Table 3:** Secondary outcomes

	All patients *n* = 50	Experimental group *N* = 25	Control group *N* = 25	*p* value
Mechanical ventilation—yes	41 (82)	25 (100)	16 (64)	< 0.001
Invasive ventilation—yes	33 (66)	17 (68)	16 (64)	
Non invasive ventilation—yes	8 (16)	8 (32)	0 (0)	
NIV failure—yes	6 (12)	6 (24)	–	–
Time on mechanical ventilation—days		14 |7–29]	22 |18–36]	0.095
Tracheostomy—yes	10 (20)	4 (16)	6 (24)	0.79
Hospital length of stay—days		27 [16–48]	26 [16–54]	0.55

Data are expressed as number (percentage) for categorical variables and as median [interquartile range] for continuous variables

The length of hospital stay was 27 [16–48] days in the experimental arm and 26 [16–54] days in the control arm (*P* = 0·79).

### Adverse events

Overall four patients (two in each study arms, *P* = 1.00) died within 90 days from randomization, of whom one died after hospital discharge (Table 4). The three hospital non-survivors had developed hospital acquired pneumonia. There were 6/25 (30%) patients who developed septic shock in the experimental arm and 5/25 (25%) patients in the control arm (*P* = 1.00). There were 7/25 (35%) patients with acute respiratory distress syndrome in the experimental arm and 6/25 (35.3%) in the control arm (*P* = 1.00). There were 0/25 (0%) patients with acute renal failure in the experimental arm, and 2/25 (10.6%) in the control group (P = 0.22). There were 1/25 (5%) patients with hepatic failure in the experimental arm and 2/25 (10%) in the control arm (P = 1.00). None of 50 patients developed hematologic failure.

**Table 4:** Distribution of serious adverse events across randomization groups

	All patients *n* = 50	Experimental group *N* = 25	Control group *N* = 25	*p*
In hospital death—yes^a^	3 (6)	1 (4)	2 (8)	1.00
Septic shock—yes	11 (22)	6 (30)	5 (25)	1.00
Acute renal failure—yes	2 (4)	0 (0)	2 (11)	0.22
Acute hepatic failure—yes	3 (6)	1 (5)	2 (10)	1.00
Acute respiratory distress syndrome— yes	13 (26)	7 (35)	6 (35)	1.00

Data are expressed as number (percentage)

^a^At 90-days post-randomization, there were four deaths, with one patient who died after being discharge alive from hospital

There was no significant difference between groups regarding the neurological scores during the entire following period (see Additional file 1: Figure S2).

## Discussion

We found that in patients with Guillain-Barré syndrome and at high risk of acute respiratory failure, early mechanical ventilation did not prevent the onset of ventilator-acquired pneumonia, resulted in higher proportion of mechanically ventilated patients. In our study, early mechanical ventilation did not decrease the incidence of pneumonia, did not prolong hospital length of stay or the time of ventilator dependency and was not associated with an increased risk of serious adverse events.

In this study, there were 29 episodes of ventilator-acquired pneumonia in 19/50 patients, with predominantly early ventilator-acquired pneumonia. These data are consistent with previous report in mechanically ventilated GBS patients [16]. Pneumonia occurred mainly as a result of subclinical swallowing impairment with subsequent aspiration precipitating invasive mechanical ventilation [15, 16]. In the current study, swallowing impairment was present at baseline in half of patients, and pathogens identified in pulmonary samples were consistent with aspiration pneumonia. A previous study suggested that delayed endotracheal intubation (> 48 h from intensive care unit admission) was a strong predictor of onset of pneumonia and arguing in favor of early mechanical ventilation [16]. However, albeit a much shorter time to mechanical ventilation (median 1 versus 5 days), as compared to controls, the incidence of ventilator-acquired pneumonia was not reduced in early mechanically ventilated patients.

Roughly 65% of patients in the control arm eventually were mechanically ventilated owing to acute respiratory failure, a proportion about three fold higher than commonly observed in unselected GBS patients [5, 6, 9]. We deliberately selected a group of patients at high risk of acute respiratory failure. Indeed, patients had the all following established risk factors for acute respiratory failure, a time from onset to admission of less than 7 days, an inability to lift the head, and a forced vital capacity of < 60% of predicted [14].

In neuromuscular diseases, the main cause of acute respiratory disease is due to hypoventilation. This hypoventilation was confirmed as a risk factor for acute respiratory failure in study by Sharshar et al. [14]. This study revealed that the vital capacity (VC) of less than 60% of the predicted value was a risk factor of acute respiratory failure. This is why in the experimental arm, we use VC and all others Sharshar’s criteria to predict acute respiratory failure. We decided to early begin mechanical ventilation with NIV if SGB patients had all Sharshar’s criteria without swallowing disorders.

In GBS patients, the use of noninvasive ventilation with bi-level airway pressure could be associated with rapid respiratory deterioration after initial improvement [22]. These findings are in contrast with the favorable effects of non-invasive ventilation in acute respiratory failure of various etiologies [23]. There are studies showing NIV is suitable in neuromuscular diseases, such as Duchenne muscular dystrophy, to treat hypoventilation without swallowing disorders and early invasive mechanical ventilation (VC between 20 and 50%) is associated with low survival [24]. In our unit, we use volume-assisted control mode in noninvasive ventilation patients. It, therefore, did not seem to us an ethical option to intubate patients without acute respiratory failure and without swallowing disorders, in other words, patients who had a good protection of the respiratory tract against inhalation. We, therefore, did not expose them at complications due to the anesthesia procedure (hypoxemia, hypotension, sedation,…). But all the patients who benefited from the NIV underwent a daily swallowing assessment, and in the event of its occurrence, intubation was performed (6 patients in our study).

In addition, in our study, only one of these eight NIV patients developed secondary pneumonia. With this low incidence in this subgroup of our study, we cannot consider NIV as a risk factor of pneumonia.

In absence of bulbar dysfunction, early noninvasive mechanical ventilation failed to prevent endotracheal intubation and there was no difference between groups in the total proportion of patients requiring invasive mechanical ventilation and in the duration of ventilator dependency. Though bulbar weakness is a major risk factor for respiratory deterioration and pneumonia in GBS patients, tools exploring subclinical swallowing impairment are lacking.

One patient refused intubation despite swallowing problems. Contrary to our protocol, it was only treated with non-invasive ventilation. He did not develop pneumonia afterwards. Since we performed an intention-to-treat analysis, we included it in our results.

In the current trial, all patients in both groups were kept in the semi-recumbent position at 30° throughout ICU stay. None of the patients received selective digestive decontamination. Though there is some evidence for a short-term benefit of selective digestive decontamination with topical and systemic antibiotics for less than 5 day [25], additional studies are needed to document the long-term effects on local sites microbial ecology and antimicrobial resistance [26]. As nasal route for the endotracheal tube may increase the risk of sinusitis and subsequent pneumonia, in both arms, all patients who required invasive mechanical ventilation were intubated via the oro-tracheal route, and cuff inflation pressure of the tube was set around 25–30 cmH20 to maintain a healthy balance between the prevention of aspiration and tracheal injury. Closed suction which may prevent ventilator-associated pneumonia has been used only in patients with refractory hypoxemia. Stress ulcer prophylaxis with proton pump inhibitor could be equally used in both arms according to national guidelines [27].

Peak cough flow was not evaluated but we analyzed maximal expiratory pressure. It is known to be correlated with forced expiratory impairment as well as peak cough flow [28]. All patients benefited daily from physiotherapy including active clearance of bronchial secretion with the same ex-insufflation technical as control arm.

The difference in the incidence between the calculation of the number of subjects required a priori, and our finding in the study, could be problematic for the power of the study. We based this calculation on the incidence published by Orlikowski et al. [16]. Between this publication and the start of inclusion in our study, the management of Guillain-Barré Syndrome has been improved (particularly with regard to the discovery of intravenous immunoglobulin). This probably explains the difference in the incidence.

Our study has several strengths, including a robust primary outcome, the probability of onset pneumonia, based on consensus definition and evaluated by three independent adjudicators who remained blinded to study interventions [29]. The recruitment period has been long. But, GBS is a rare condition. However, we are the reference center in our country for this type of disease, it seems difficult to produce the same study with a shorter time.

The trial was conducted at one single referral center and included a highly selected population at high risk of acute respiratory failure. Thus, the trial generalizability may be limited. Study treatments could not be masked to the ICU staff and investigators as they had to take care of respiratory management.

In conclusion, in the present study including adults with Guillain-Barré syndrome at high risk of respiratory failure, we did not observe a prevention of pneumonia with early mechanical ventilation.

## Supplementary information

Additional file1 (DOCX 129 kb) **Figure S1. **Cumulative incidence of tracheostomy. **Figure S2.** Neurological scores during the entire following period. **Table S1. **Predominant organisms in early and late-onset pneumonia^*^. **Table S2. **Secondary outcomes. **Table S3. **Distribution of serious adverse events across randomization groups^*^.

## Data Availability

The datasets supporting the conclusions of this article are included within the article and its additional file.

## Abbreviations

EMV: Early mechanical ventilation
FVC: Forced vital capacity
GBS: Guillain-Barré syndrome
IvIg: Intravenous Immunoglobulin
MRC sumscore: Medical Research Council sumscore
MIP: Maximal inspiratory pressure
MEP: Maximal expiratory pressure
PaCO_2_: Arterial pressure of carbon dioxide
PaO_2_: Arterial pressure of oxygen
SAPSII: Simplified acute physiology score II
SatO_2_: Transcutaneous saturation of oxygen
