# Supplementary material for: Early mechanical ventilation in patients with Guillain-Barré syndrome at high risk of respiratory failure: a randomized trial
Source: Ann Intensive Care. 2020 Sep 30;10:128. doi: 10.1186/s13613-020-00742-z (PMC7525233; doi:10.1186/s13613-020-00742-z)
Supplement: Supplementary file 1 — Additional file1 (DOCX 129 kb) Figure S1. Cumulative incidence of tracheostomy. Figure S2. Neurological scores during the entire following period. Table S1. Predominant organisms in early and late-onset pneumonia*. Table S2. Secondary outcomes. Table S3. Distribution of serious adverse events across randomization groups*. [file 13613_2020_742_MOESM1_ESM.docx]

Appendix

**
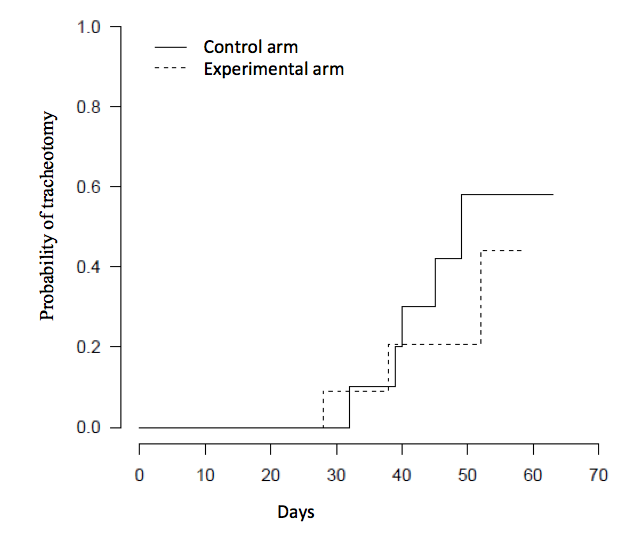
**

**Figure S1. Cumulative incidence of tracheostomy**


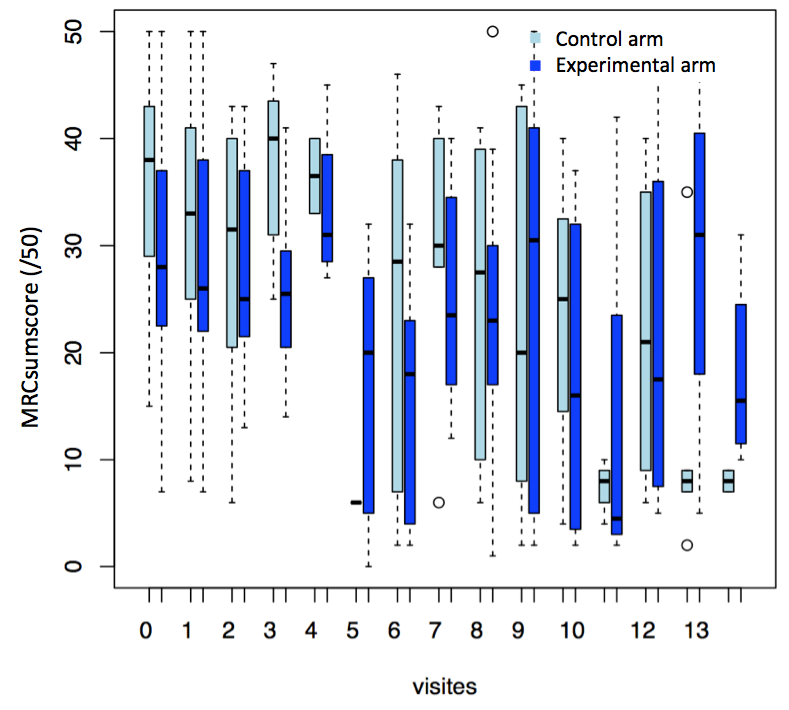


**Figure S2. Neurological scores during the entire following period**

**Table S1: Predominant organisms in early and late-onset pneumonia***

|  | | **Early-onset pneumonia** | **Late-onset pneumonia** |
| --- | --- | --- | --- |
| Type of respiratory sample | Polymicrobien | 9 (41%) | 14 (64%) |
|  | Monomicrobien | 9 (41%) | 7 (32%) |
|  | Negative | 4 (18%) | 1 (4%) |
| Culture of respiratory sample | *Haemophilus influenzae* | 8 (36%) | 3 (14%) |
|  | methicillin-susceptible *Staphylococcus aureus* | 8 (36%) | 14 (63%) |
|  | *Streptococcus pneumonia* | 4 (18%) | - |
|  | Other *Streptococci* | 3 (14%) | - |
|  | *Pseudomonas aeruginosa* | 3 (14%) | 13 (60%) |
|  | *Klebsiella pneumoniae* | - | 4 (18%) |
| Total | | 22 | 22 |

*Data are expressed as number (percentage).

**Table S2. Secondary outcomes ^*^**

|  | **All patients**  **n=50** | **Experimental group**  **N=25** | **Control group**  **N=25** | **p value** |
| --- | --- | --- | --- | --- |
| Mechanical ventilation -yes | 41 (82) | 25 (100) | 16 (64) | <0.001 |
| Invasive ventilation- yes | 33 (66) | 17 (68) | 16 (64) |  |
| Non invasive ventilation- yes | 8 (16) | 8 (32) | 0 (0) |  |
| NIV failure- yes | 6 (12) | 6 (24) | - | - |
| Time on mechanical ventilation- days |  | 14 \|7-29] | 22 \|18- 36] | 0.095 |
| Tracheostomy - yes | 10 (20) | 4 (16) | 6 (24) | 0.79 |
| Hospital length of stay - days |  | 27 [16-48] | 26 [16-54] | 0.5 |

*Data are expressed as number (percentage) for categorical variables and as median [interquartile range] for continuous variables.

**Table S3. Distribution of serious adverse events across randomization groups^*^**

|  | **All patients**  **n=50** | **Experimental group**  **N=25** | **Control group**  **N=25** | ***p*** |
| --- | --- | --- | --- | --- |
| In hospital death- yes† | 3 (6) | 1 (4) | 2 (8) | 1.00 |
| Septic shock- yes | 11 (22) | 6 (30) | 5 (25) | 1.00 |
| Acute renal failure- yes | 2 (4) | 0 (0) | 2 (11) | 0.22 |
| Acute hepatic failure-yes | 3 (6) | 1 (5) | 2 (10) | 1.00 |
| Acute respiratory distress syndrome- yes | 13 (26) | 7 (35) | 6 (35) | 1.00 |

*Data are expressed as number (percentage).

†at 90-days post randomization there were 4 deaths, with one patient who died after being discharge alive from hospital
